# Dental Considerations in the Management of Glanzmann’s Thrombasthenia

**DOI:** 10.5005/jp-journals-10005-1054

**Published:** 2010-04-15

**Authors:** Diana N Mehta, Rupinder Bhatia

**Affiliations:** 1 Postgraduate Student, Department of Pediatric and Preventive Dentistry, Padmashree Dr. DY Patil Dental College and Hospital Navi Mumbai, Maharashtra, India; 2 Professor and Head, Department of Pediatric and Preventive Dentistry, Padmashree Dr. DY Patil Dental College and Hospital Navi Mumbai, Maharashtra, India

**Keywords:** Glanzmann’s thrombasthenia, platelet disorders, pediatric dentist, “Acrylic-splint” appliance.

## Abstract

Glanzmann’s thrombasthenia, is one of the rarest congenital, genetically inherited platelet disorder. It has an incidence of about 1:1,000,000, but is more common in populations with increased consanguinity. Glanzmann’s thrombasthenia is characterized by deficiency or dysfunction of glycoprotein (GP) lib and Ilia, which are the receptors of fibrinogen. Both sexes are equally affected.

Typical mucocutaneous bleeding occurs at birth or early infancy. Obtaining appropriate dental history of excessive bleeding after dental extraction, unexplained spontaneous mucocutaneous bleeding, gingival bleeding during teething or shedding of deciduous teeth and petechiae, ecchymoses or purpura on mucous membranes can play an important part in diagnosis. Hence, the pediatric dentist plays a very crucial role for prompt diagnosis and management of Glanzmann’s thrombasthenia.

Presenting here is a known case of Glanzmann’s thrombasthenia, of a 6-year-old girl who required to undergo dental extraction and its successful management using an “acrylic-splint” along with the placement of “Calgigraf-Ag Foam”.

## INTRODUCTION

Glanzmann’s thrombasthenia, is one of the rarest congenital, genetically inherited platelet disorder.^1-4^ The gene responsible is carried on the long-arm of chromosome 17 at q in humans, one of the twenty-two pairs of autosomal chromosomes, so it affects male and female equally.^2^ Dr Eduard Glanzmann, a German pediatrician, first described this bleeding disorder in children from a village in the Swiss Alps. Only 130 cases were reported worldwide till 1969.^5^ Glanzmann thrombasthenia has an incidence of about 1:1,000,000, but is more common in populations with increased consanguinity.^4^

Glanzmann’s thrombasthenia is characterized by deficiency or dysfunction of glycoprotein (GP) IIb and IIIa, which are the receptors of fibrinogen. Hence, no fibrinogen bridging can occur and bleeding time is significantly prolonged, clot retraction is diminished and platelets do not aggregate during blood coagulation or after addition of ADP (adenosine diphosphate).^6^

Glanzmann’s thrombasthenia is classified into three types as given by Caen in 1972, depending on the level of GPIIb-IIIa present:

- Type 1 (severe) : < 5% of normal GPIIb-IIIa levels.
- Type 2 (less severe): 10 to 20% of normal GPIIb-IIIa levels.
- Type 3 (variant): Normal levels of GPIIb-IIIa, but functionally inactive.

The clinical severity of Glanzmann’s thrombasthenia does not correlate with the subtype.^7^

Excessive bleeding after dental extraction may often be the first sign. Children with Glanzmann’s thrombasthenia are often diagnosed early in life, and often before the age of five, usually by unexplained spontaneous mucocutaneous bleeding. Life expectancy is normal. Bleeding episodes in people with Glanzmann’s thrombasthenia include: Nose bleeds, easy bruising, gingival bleeding during teething or shedding of deciduous teeth during childhood or even over-vigorous tooth brushing, purpura, gastrointestinal bleeding, CNS hemorrhage, hematuria, muscle hematoma, hemarthrosis and menorrhagia.^3,8^ Hence, the pediatric dentist should record case history carefully for proper diagnosis and treatment planning.

## CASE HISTORY

**Fig. 1:**
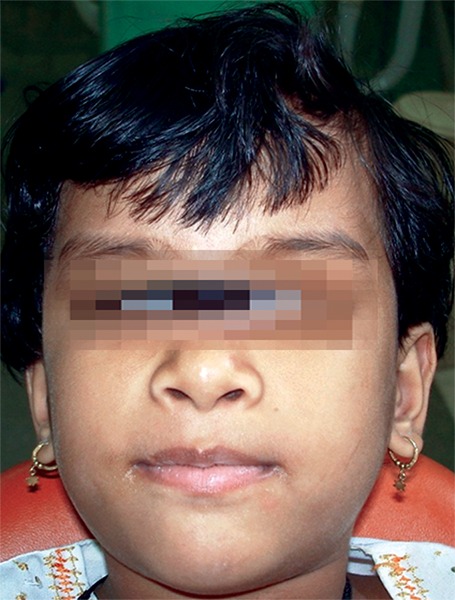
Girl diagonosed with Glanzmann’s thrombasthenia

A 6-year-old girl (Fig. 1) reported to the Department of Pediatric and Preventive Dentistry with a chief complaint of pain in upper left back region of jaw (65) since 2 to 3 months. Her past medical history revealed that she was a known case of Glanzmann’s thrombasthenia since age of three years with a history of epistaxis since 6 months of age. She was operated for CNS hemorrhage 2 years back and was given platelet transfusion during same. She had a seizure disorder since 1 year and also had history of spontaneous ecchymoses and easy bruising. Proper immunization schedule was followed and milestones were normal.

Past laboratory tests revealed normal platelet counts and morphology, prolonged bleeding times, decreased clot retraction, and abnormal platelet aggregation responses to physiologic stimuli.

Her family history revealed that there was consanguineous marriage of parents, but no other family member had suffered from similar problem.

**Fig. 2:**
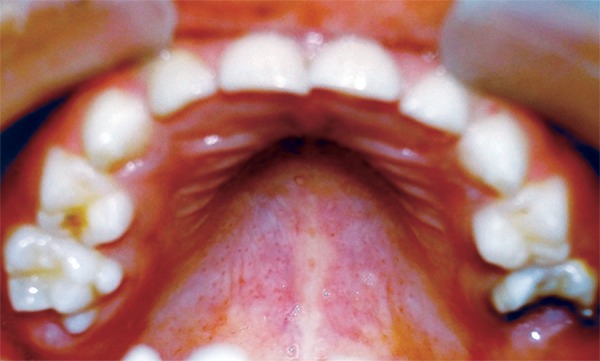
Maxillary occlusal photograph showing grossly destructed 65

**Fig. 3:**
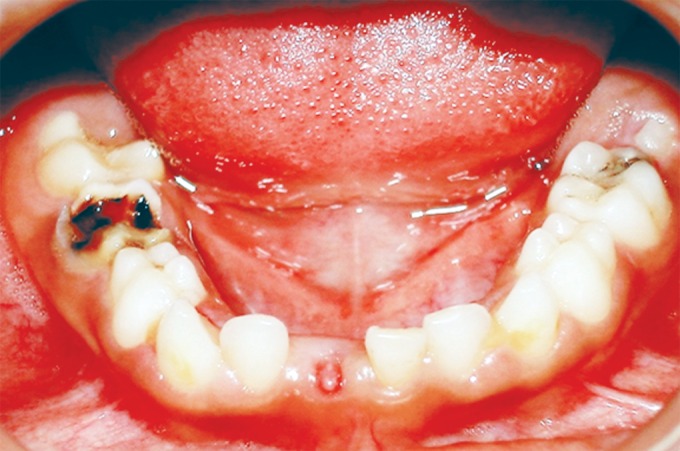
Mandibular occlusal photograph showing proximal caries on 75 and arrested grossly carious 85

**Fig. 4:**
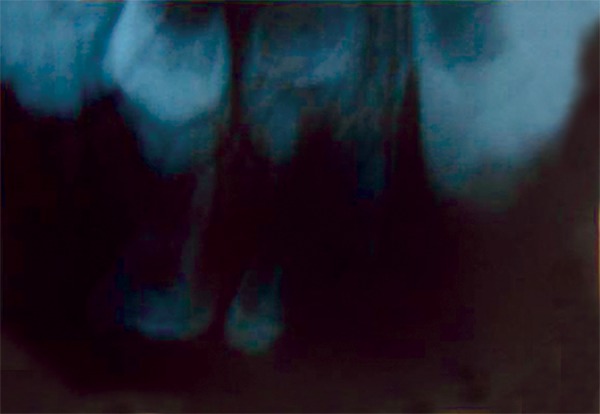
IOPA of 65 showing radiolucency involving enamel dentin and pulp with severe periradicular bone loss

**Figs 5A and B:**
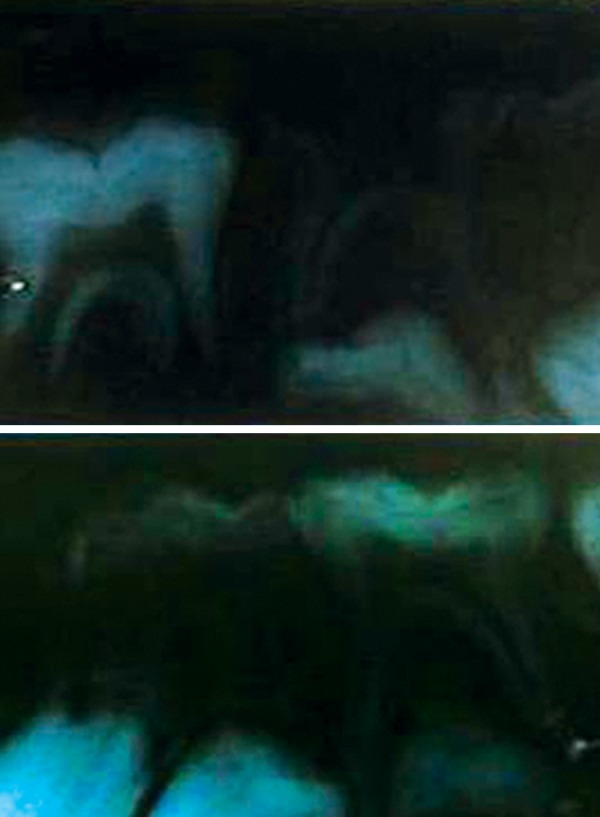
IOPA of 75 and 85 showing radiolucency involving enamel and dentin

On examination the child was conscious, cooperative (Rating 2, on Frankl Behavior Rating Scale) with normal gait and was well-oriented. Intraoral examination showed grossly destructed 65 (Fig. 2), proximal caries on 75 and arrested grossly caries with 85 (Fig. 3). Intraoral periapical radiograph of 65 showed radiolucency involving enamel, dentin and pulp with severe periradicular bone loss (Fig. 4) and of 75 and 85 showed radiolucency involving enamel and dentin (Figs 5A and B).

**Fig. 6:**
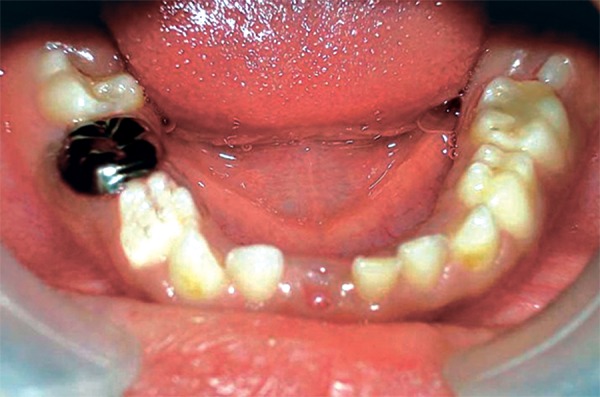
85 restored with a stainless steel crown and 75 with composite

Diet analysis, counseling and oral prophylaxis were done. 75 was restored with composite. After a build-up of 85 crown structure with glass ionomer cement, a stainless steel crown was given (Fig. 6). Use of suction tip was avoided for isolation to prevent any bruising of oral mucosa and cotton rolls were used instead.

**Fig. 7:**
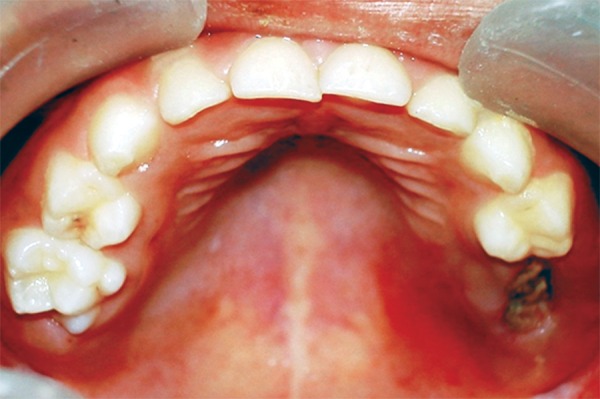
Healing of socket satisfactory after 7 days

After consultation with a pediatrician, appropriate medical care measures were taken and the child was admitted to the hospital. Under prophylactic antibiotics 65 was extracted using local anesthesia (containing 2% lidocaine with 1:100,000 epinephrine) infiltration. Hemostasis was achieved by compression of socket and ice pack application. The patient was given postoperative instructions and was discharged from the hospital after the pediatrician’s consent. The patient was recalled to evaluate healing of socket after 1 day and 7 days which was satisfactory (Fig. 7).

**Fig. 8:**
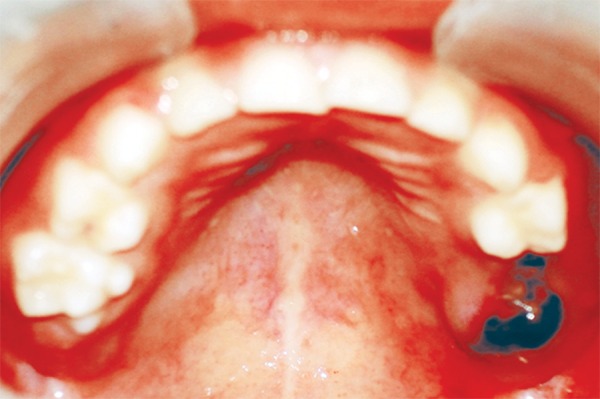
“Liver-clot” seen in socket of 65

**Fig. 9:**
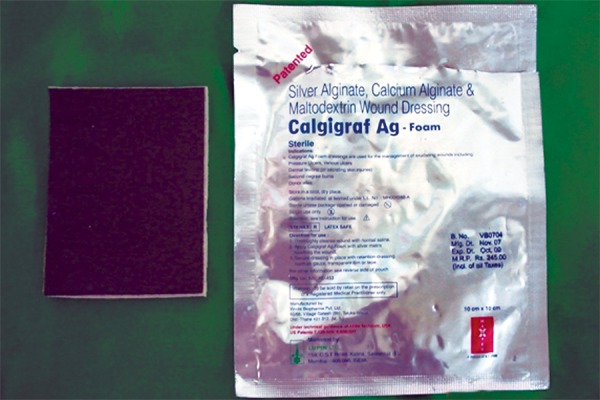
Calgigraf AG-foam

Ten days postextraction the child reported back to the department with complaint of bleeding from socket which started on eating some hard food. On examination of the socket there was dislodgement of fibrous clot and presence of a “liver-clot” (Fig. 8). On discussion with the pediatrician, the placement of “Calgigraf Ag-Foam” (Viridis Biopharma Pvt. Ltd., Mumbai) (Fig. 9) into socket was done and the fabrication of an ’acrylic-splint’ appliance with the following objectives was done:

1. To aid in retention of the “Calgigraf Ag-Foam” in the extraction socket.
2. To act as a splint in preventing any damage to healing wound.
3. To serve as a space maintainer.

**Fig. 10:**
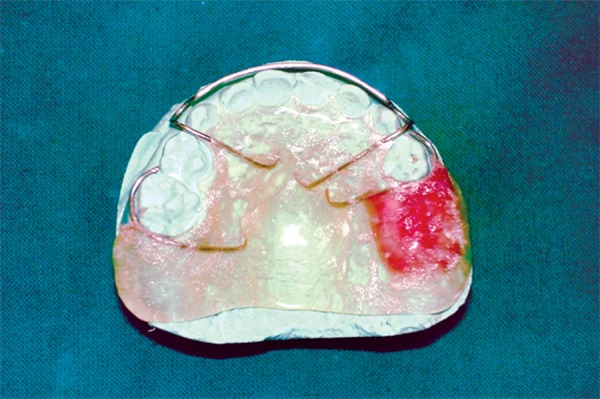
Fabricated acrylic splint appliance

The following steps were carried out prior to the delivery of the appliance thus fabricated:

1. Education and motivation of child and parent were done regarding taking proper precautions to prevent any damage to healing socket.
2. Oral prophylaxis was done. An impression was made of the maxillary and mandibular arches using alginate material. In order to familiarize the child with the impression procedure, the mandibular impression was taken first.
3. Impressions were poured in dental stone and spacer wax was adapted to the socket region to provide sufficient relief from any pressure from appliance.
4. Two C-clasps were fabricated using orthodontic wire over teeth- 55 and 64, to aid in retention.
5. After applying separating medium, sprinkle-on technique was used to make a clear “Acrylic-splint” appliance.
6. Finishing and polishing of “Acrylic-splint” appliance was done and thus was ready for delivery (Fig. 10).

**Fig. 11:**
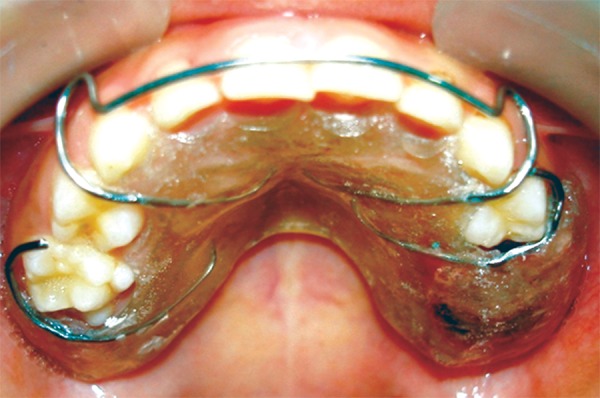
Appliance positioned in the child’s mouth

**Fig. 12:**
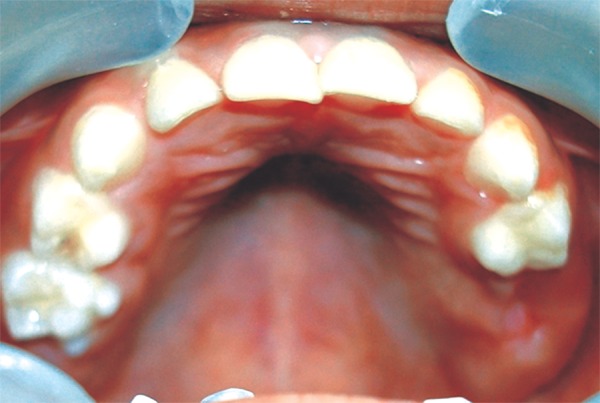
Healing satisfactorily complete at 2 month recall visit

The appliance was positioned in the child’s mouth and areas of excessive pressure on intraoral tissues were identified and reduced (Fig. 11). Instructions were given to parents and child regarding oral hygiene maintenance, placement and daily care of the appliance. A recall visit was scheduled after two days of appliance-delivery for further assessment. Monthly follow-ups were planned to assess healing of extraction socket and eruption of 26. At 1 month recall healing was slow and inadequate. At the 2-month recall visit healing was satisfactorily complete (Fig. 12). Oral prophylaxis and fluoride application was done. Patient was recommended to continue wearing the appliance until the eruption of 26, which was expected to be soon, as eruption bulge was present. After eruption of 26 the present appliance will be replaced by a reverse band and loop space maintainer.

## DISCUSSION

The pediatric dentist plays a very crucial role in diagnosis of Glanzmann’s thrombasthenia in evaluating dental history of excessive bleeding after dental extraction, unexplained spontaneous bleeding from mucous membranes, gingival bleeding during teething or shedding of deciduous teeth and petechiae, ecchymoses or purpura on mucous membranes. The differential diagnosis includes von Willebrand’s disease, Bernard-Soulier syndrome and platelet secretory defects.^8^ In the present case, the child’s past medical history revealed that she was a known case of Glanzmann’s thrombasthenia since age of three years with a history of epistaxis since 6 months after birth. She had been operated for CNS hemorrhage 2 years back and was given platelet transfusion during same. She had a seizure disorder since 1 year and also had history of spontaneous ecchymoses and easy bruising.

Laboratory test of patients with Glanzmann’s throm-basthenia shows prolonged bleeding times, decreased or absent clot retraction, and abnormal platelet aggregation responses to physiologic stimuli, according to literature because the platelets of these patients have a normal initial slope of ristocetin-induced aggregation, reflecting the normal levels of plasma von Willebrand factor and the normal platelet GPIb/IX content; the reduced second wave of aggregation at low doses of ristocetin reflects the impaired GPIIb-IIIa function, and the interesting cyclical aggregation at higher doses of ristocetin probably reflects a complex interaction between ristocetin-induced binding of von Willebrand factor to GPIb/IX and inhibition of this interaction by released ADP. In each case, the abnormalities reflect the inability of the platelets to bind fibrinogen and/or other adhesive glycoproteins.^6^ In the present case, past laboratory tests revealed normal platelet counts and morphology, prolonged bleeding times, decreased clot retraction, and abnormal platelet aggregation responses to physiologic stimuli.

According to literature, a cure for the disease does not exist; the only effective therapy consists of transfusions of fresh platelets or platelet concentrates.

- Other treatments include compression, gelatine sponge or gauze, antifibrinolytic agents such as tranexamic acid or topical thrombin and YAG laser can be used to control minor bleeding.
- Desmopressin (DDAVP) has been tried in some patients with Glanzmann’s thrombasthenia and may shorten bleeding time in patients with type 2 only, but there is no notable clinical efficacy.
- Oral contraceptives can regularize menstrual cycles and reduce the bleeding. This is sometimes recommended before a girl’s first period, as hemorrhage is particularly severe at this time
- Immunoabsorption is the removal of antibodies to platelets by plasma exchange with the use of protein-A sepharose columns which may transiently restore platelet efficacy. However, this technique is not available everywhere, it is labor intensive, and requires an adequate venous access. It is not of use in control of active bleeding as this process requires several hours.
- Allogenic marrow transplant has been reported in two patients successfully with Glanzmann’s thrombas-thenia.^1,3,6,8^

In the present case, dental management was done by compression of socket and ice pack application immediately after extraction and hemostasis was achieved. Use of suction tip and high vacuum pressure was avoided to prevent any damage to oral mucosa and local anesthesia contained 2% lidocaine with 1:100,00 epinephrine, which is a vasoconstrictor and aids in hemostasis.

A “liver clot” is typically dark red in color and usually protrudes from the surgical site. It is not prudent to protect a loose clot according to literature.^9^ Douglas J Sanders (1958) was the first to discuss about the fabrication of acrylic space maintainer. In the present case the liver clot was dislodged and the socket was packed with “Calgigraf Ag-Foam” (Viridis Biopharma Pvt. Ltd., Mumbai), with the following composition: Silver alginate, calcium alginate and maltodextrin wound dressing. To aid in retention of this dressing, a nonfunctional, passive, acrylic removable splint which also served as space maintainer was fabricated.

## CONCLUSION

Glanzmann’s thrombasthenia, is one of the rarest congenital, genetically inherited platelet disorder. The pediatric dentist plays a very crucial role in prompt diagnosis of Glanzmann’s thrombasthenia by evaluating the dental history and may be the first to diagnose the disorder. The pediatric dentist also plays an important role in advising and maintaining good oral hygiene and periodic oral assessment, thereby preventing any dental health problems and providing an improved quality of life for the patient.
